# Oral health status of adults in Southern Vietnam - a cross-sectional epidemiological study

**DOI:** 10.1186/1472-6831-10-2

**Published:** 2010-03-13

**Authors:** Thoa C Nguyen, Dick J Witter, Ewald M Bronkhorst, Nhan B Truong, Nico HJ Creugers

**Affiliations:** 1 Department of Prosthodontics, Dental School, University of Medicine and Pharmacy, Can Tho, Vietnam; 2 Department of Oral Function and Prosthetic Dentistry, College of Dental Science, Radboud University Nijmegen Medical Centre, Nijmegen, The Netherlands; 3 Department of Preventive and Restorative Dentistry, College of Dental Science, Radboud University Nijmegen Medical Centre, Nijmegen, The Netherlands; 4 Department of Epidemiology, Faculty of Public Health, University of Medicine and Pharmacy, Can Tho, Vietnam

## Abstract

**Background:**

Before strategies or protocols for oral health care can be advised at population level, epidemiological information on tooth decay patterns and its effects on oral function are indispensable. The aim of this study was to investigate influences of socio-demographic variables on the prevalence of decayed, missing, filled (DMF) and sound teeth (S_t_) and to determine the relative risk of teeth in different dental regions for D, M, and F, of adults living in urban and rural areas in Southern Vietnam.

**Methods:**

Cross-sectional DMF and S_t _data of 2965 dentate subjects aged 20 to 95 living in urban and rural areas in three provinces were collected by means of a self-administered questionnaire and an oral examination. The sample was stratified by age, gender, residence and province.

**Results:**

The percentage of subjects having missing teeth was high for all ages while it was low for subjects with decayed and filled teeth. The mean number of missing teeth increased gradually by age from approximately 1 in each jaw at the age of 20 to 8 at the age of 80. The number of decayed teeth was relative low at all ages, being highest in molars at young ages. The mean number of filled teeth was extremely low at all ages in all dental regions. Every additional year of age gives a significantly lower chance for decay, a higher chance for missing, and a lower chance for filled teeth. Molars had a significantly higher risk for decay, missing and filled than premolars and anterior teeth. Females had significantly higher risk for decayed and filled teeth, and less chance for missing teeth than males. Urban subjects presented lower risk for decay, but approximately 4 times greater chance for having fillings than rural subjects. Low socio-economic status (SES) significantly increased the chance for missing anterior and molar teeth; subjects with high SES had more often fillings.

**Conclusions:**

The majority of adults of Southern Vietnam presented a reduced dentition. The combination of low numbers of filled teeth and relative high numbers of decayed and missing teeth indicates that the main treatment for decay is extraction. Molars are more at risk for being decayed or missing than premolars and anterior teeth.

## Background

Little published information is available about the oral health status of the population of Vietnam. The National Oral Health Survey of Vietnam 2001 showed that the prevalence of caries amongst adults in Vietnam is high [1]. According to this survey, the numbers of decayed/missing/filled teeth (DMFT) in subjects aged 45 years and over ranged from 6.09 to 11.66 in different regions of Vietnam. The high numbers of missing teeth (4.45 to 8.59 for those aged 45 years and over) combined with the low numbers of filled teeth (0.02 to 0.36) indicate that extraction was the most common treatment for caries and that restorative treatment was low. As a result, large parts of the population of Vietnam must have incomplete natural dentitions.

It has been demonstrated that the impact of missing teeth on oral functions and quality of life is dependent on location and type [2]. Generally, absent molars are considered to have less impact on oral functions and quality of life than absent anterior teeth [3-5]. Based on this knowledge, concepts like the "shortened dental arch" have been developed as a possible solution in situations where dental services are limited or unaffordable. This concept is a strategy that aims to preserve adequate oral function by focusing dental resources on the anterior and premolar teeth and to avoid complex restorative treatment in the molar area. In a review paper of the feasibility of the shortened dental arch concept has been demonstrated for several countries, including a low-income country (Tanzania) [6,7].

Vietnam is also a low-income country with 72% of the population living in rural areas [8]. The most recent data indicated a total health expenditure of US $57.5 per capita in the year 2007, which is 7.1% of GDP [9]. Of this expenditure, the government provides only 8.7%; the remaining part of the cost is paid by the population [9]. The costs are high, even compared with other low and middle-income countries [10]. In addition, there are insufficient dental resources in Vietnam. Most dental practices are located in urban areas and only few rural residents have access to any dental health services [11]. This is predominantly true in Southern Vietnam. In 2008, the population of Southern Vietnam was about 45 million while there were about 850 active dentists, 400 denturists, and 800 dental nurses in the governmental dental care system [12]. On average the ratio of dentists to the general population in this region is 1/43,000, ranging from 1/178,500 in rural areas to 1/13,400 in urban areas. Notably, in 156 rural districts (of 363 rural districts) there are no dentists at all [12].

The importance of appropriate oral health care strategies and adequate tailor made intervention protocols in dealing with these limitations is widely recognized [13-15]. However, before strategies or protocols such as the shortened dental arch concept can be advised at population level, epidemiological information on tooth decay patterns and its effects on oral function are indispensable. The present cross-sectional descriptive study is the first of a series in finding and analyzing this information for the Southern Vietnamese population. The aim of this study was to determine the oral health status of adults living in urban and rural areas in Southern Vietnam and to analyze the influence of several socio-demographic variables. A second aim was to analyze the relative risk of teeth located in different dental regions by means of decayed, missing, filled (DMFT) and sound teeth.

## Methods

### Sample construction

A cluster stratified sampling design was used to draw 4050 subjects aged ≥ 20 years from urban and rural areas of three provinces in Southern Vietnam: Cantho, Angiang and Hochiminh (HCM). The three provinces were chosen because they were considered representative for the population of Southern Vietnam. Data for this study were collected between 2006 and 2009.

For the urban sample, in each province, subjects were obtained from 5 factories or institutions with more than 300 employees. These factories and institutions were chosen because of accessibility. A complementary sample was drawn in neighbourhoods adjacent to each factory or institution because no employees of 60 and over could be found in the factories or institutions. Subjects were randomly selected from lists of employees and administrative lists of citizens obtained from local authorities (Table 1).

**Table 1:** Number of selected factories or institutions (with adjacent neighbourhoods) and number of selected subjects in the urban and rural areas of the three provinces Cantho, Angiang, and HCM

	Urban		Rural		
		
Province	Factories/Institutions	Number of subjects aged ≥ 20	Communes	Number of subjects aged ≥ 20	Total sample
Cantho	5	957	7	707	1664
Angiang	5	564	9	631	1195
HCM	5	591	5	600	1191

Total	15	2112	21	1938	4050

For the rural sample, all rural districts of the three provinces were included in the study. In each rural district, one rural sub-district was randomly chosen. In each selected sub-district, one commune (a lower administrative level of rural sub-districts) was selected for reasons of accessibility. Subjects were randomly selected from the villagers using administrative lists of local authorities (Table 1).

The inclusion aimed an equal distribution of subjects according to residence (urban, rural), province (Cantho, Angiang and HCM), gender, and age groups (20-29, 30-39, 40-49, 50-59, and over 60 years). Of the 4050 subjects invited to participate, 977 subjects (24%) did not participate for reasons including no permission to leave from work (561), travel (181), refusal (73), moving to another area (49), illness (30), and other various reasons (59) (i.e. administrative failure). Twenty-four subjects with acute oral infections and subjects who were not capable of responding either for physical or mental reasons were also excluded. As a result, 3073 subjects were included (Table 2).

**Table 2:** Number of participants and response rate (%) of invited subjects per province

Provinces				
	**Cantho**	**Angiang**	**HCM**	**Total**
Residence				
Urban	703 (73)	446 (79)	412 (70)	1555 (74)
Rural	538 (76)	556 (88)	418 (70)	1518 (78)
Gender				
Male	603 (75)	495 (78)	384 (65)	1482 (73)
Female	638 (77)	507 (80)	446 (75)	1591 (77)
Total	1242 (75)	1002 (84)	830 (70)	3073 (76)

### Questionnaire and clinical examination

After obtaining verbal consent, subjects were asked to complete a self-administered questionnaire including a number of background variables. For the present study, data regarding age, gender and socio-economic status (SES) were collected. In case subjects were illiterate or unable to read because of visual impairment, a dental assistant read aloud the questions and recorded the answers.

For each subject, SES was assessed using the (slightly modified) Kuppuswamy classification [16]. This classification has been developed for low-income populations and is based on a subject's level of education, occupation, and household income [16,17]. An SES scale (high, middle, low) was constructed according to the summed score of each variable (Table 3).

**Table 3:** Constructed socio-economic status (SES) classification (modified Kuppuswamy classification)

***Level of Education***	**Assigned score**
Higher education (level ≥ 12*)	6
College (level 6 to 12*)	5
Primary school (level 1 to 5*)	3
Informal education (i.e. self learning); literate	2
No formal education	1
*Occupation*	
Professional and skilled	4
Business, household keeper	3
Retired/unemployed	2
Semiskilled and unskilled	1
*Household income*	
Income covers expenses; no loans needed	4
Income does not cover expenses; no loans needed	3
Income covers expenses; loans needed incidentally	2
Income does not cover expenses; loans needed regularly	1
*Socio-economic status scale*	*Summed score*
High	9 - 14
Middle	7, 8
Low	3 - 6

*Education levels based on the Vietnamese classification system

Following the completion of the questionnaires, subjects received an oral examination. Of the variables recorded, only tooth status (decayed, missing, filled, and sound) was used for the present analyses. Other variables (e.g. tooth wear, bleeding on probing, and tooth replacements) were not considered. Following WHO criteria [18], a tooth was considered 'decayed' if primary caries was detected, if the tooth was fractured, or if the tooth was filled but showed secondary caries. Caries was assessed by visual inspection, with additional tactile inspection with a dental probe if required. Only cavitated lesions with softened surfaces were recorded as caries. In case of doubt, no caries was recorded. In addition, a tooth root was recorded as 'decayed'. A tooth was recorded as 'missing' if the tooth was clinically absent. 'Filled' was recorded for teeth having a dental restoration without the presence of caries. Finally, present teeth without being decayed or filled were considered 'sound' (S_t_).

One calibrated examiner performed the examinations in natural light using a mirror and a dental probe, with the subjects seated in an ordinary chair. A headlight was used when the natural light was felt to be insufficient. The research was carried out in compliance with the Helsinki Declaration. The Educational Scientific Committee of Cantho University of Medicine & Pharmacy granted ethics approval for this study.

### Data analyses

Edentulous subjects were excluded from the analyses of D, M, and F. Because the number of people in the age of 81 year and older was relatively small (less than 10 persons), analyses were restricted to subjects with a maximum age of 80.

To determine the scores of decayed, missing, filled and sound teeth for the whole mouth, and for the three dental regions by age, the average number of D, M, F and S_t _per subject was plotted against age for the whole dentition and for the anterior, premolar and molar regions separately.

Multivariate regression analyses were performed separately for upper and lower jaws to determine the effects of age, gender, residence, province and SES on the distribution of non-sound teeth over D, M and F, i.e.  (D_ratio_),  (M_ratio_), and  (F_ratio_). Since these distributions were very skewed, the ratios were dichotomized using the following cut-off points: for D_ratio _(decay present; no decay), M_ratio _(all non-sound teeth missing; not all non-sound teeth missing), F_ratio _(fillings present; no fillings present). Codings for the independent variables in the final logistic regression models were: age (numerical variable), gender, residence, province (Angiang *vs *Cantho, HCM *vs *Cantho), and SES (SES_high _*vs *SES_middle_, SES_low _*vs *SES_middle_).

The relative D, M, F and S_t _scores per dental region (D_rel_, M_rel_, F_rel _and S_trel_) were determined by dividing the number of teeth having the respective status by the total number of teeth concerned in each region. Mean D_rel_, M_rel_, F_rel _and S_trel _of each dental region were compared by paired T-tests.

## Results

Of the 3073 subjects selected, 108 (3.5%) were edentulous, of which 91 subjects were older than 60 years. The remaining 2965 subjects were included in the analyses.

Logistic regression analyses were performed separately for upper and lower jaws. It appeared that effects, if present, were always in the same direction. Therefore, regression analyses of upper and lower jaws were combined.

### Decayed teeth

**Figure 1:**
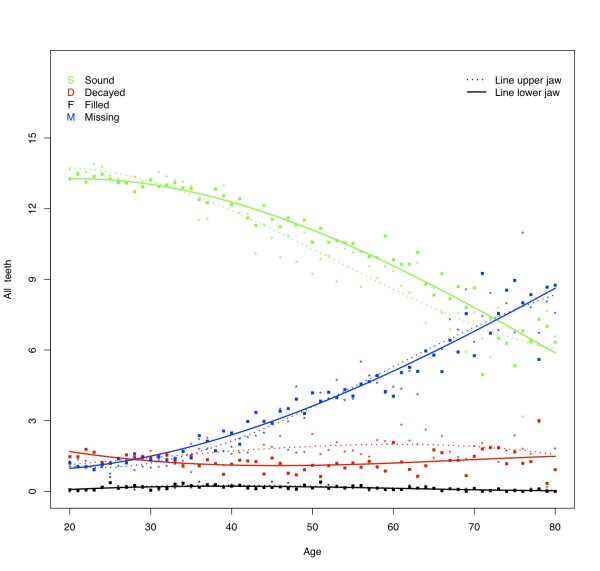
**Mean number of decayed (D), missing (M), filled (F), and sound (S_t_) teeth by age**.

**Figure 2:**
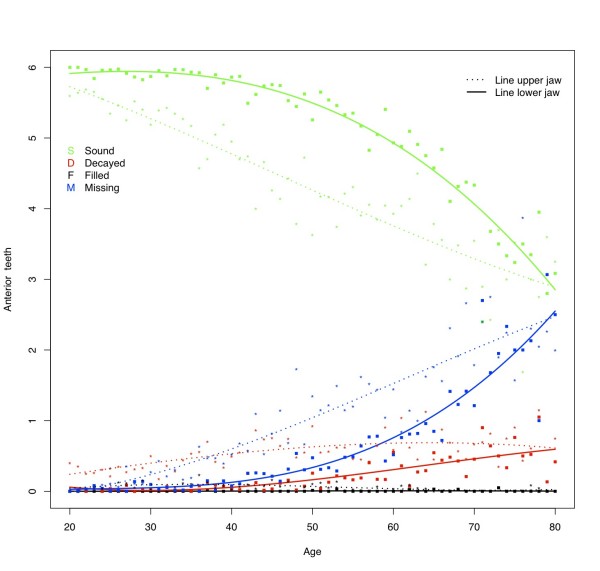
**Mean number of decayed (D), missing (M), filled (F), and sound (S_t_) teeth in the upper and lower anterior region by age**.

**Figure 3:**
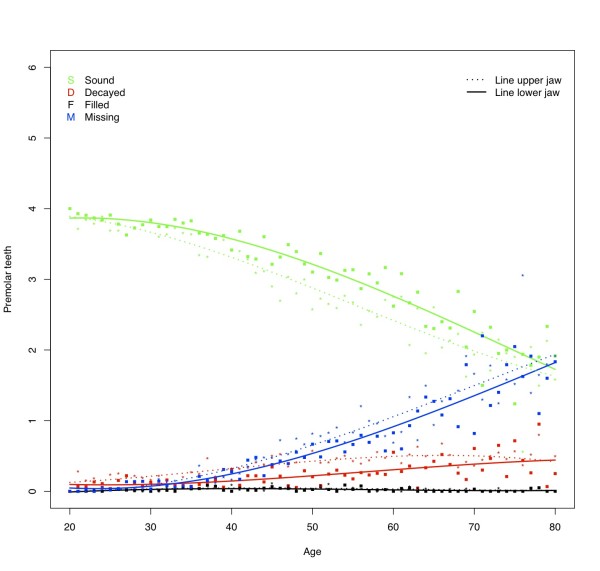
**Mean number of decayed (D), missing (M), filled (F), and sound (S_t_) in the upper and lower premolar region by age**.

**Figure 4:**
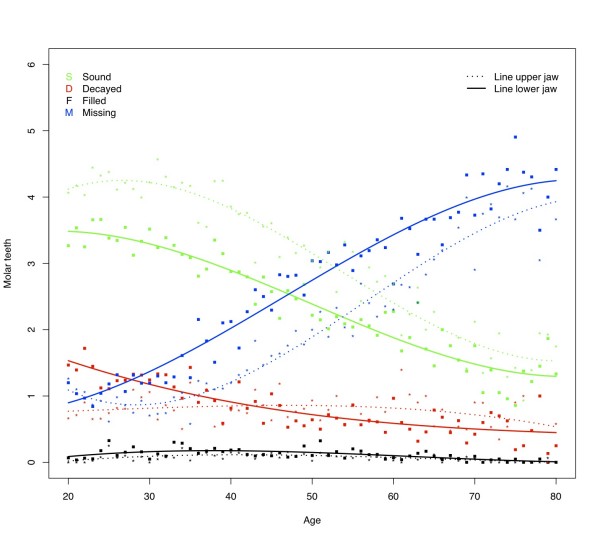
**Mean number of decayed (D), missing (M), filled (F), and sound (S_t_) in the molar region by age**.

The percentage of subjects having one or more decayed teeth was found to be approximately 30% (Table 4). At all ages subjects in this sample have on average approximately 1.5 decayed teeth in each jaw (Figure 1). From 25 years of age, the D component for the upper jaw is slightly higher than for the lower jaw. This trend can be seen in all dental regions (Figures 2 and 3) except in the molar region (Figure 4), where the D component for the lower jaw is relatively high in subjects under the age of approximately 40 years. At younger ages the number of decayed teeth is highest in the molar region compared to the anterior and premolar regions. However, from the age of approximately 40 years this difference is diminishing (Figures 2, 3 &4).

**Table 4:** Percentage of subjects with decayed, missing, and filled teeth according to age category and residence

	Number of subjects	Percentage of subjects with		
		**Decayed teeth**	**Missing teeth**	**Filled teeth**
Age group				
20-29	509	32	70	15
30-39	607	33	78	18
40-49	619	28	91	17
50-59	676	26	92	15
≥ 60	554	30	96	8
Residence				
Urban	1381	32	87	24
Rural	1584	28	85	6

The regression analysis (Table 5) shows that for all three dental regions each additional year of age gives a significantly lower chance for having decayed teeth in each region (OR: 0.979 - 0.988). However, for the whole dentition the influence of age on decay could not be demonstrated. Females have a higher risk (OR: 1.37) for having decayed teeth. This relationship is most prominent for the premolar region (OR: 1.52, *P *< 0.001), less prominent for the molar region (OR: 1.21, *P *< 0.05), and not found for the anterior region. Urban subjects have a significantly lower risk for having decayed teeth compared to rural subjects. With respect to province, subjects living in HCM have less risk for having decayed teeth than subjects living in the other provinces.

**Table 5:** Odds ratios of dichotomized D, M, and F ratios, 95% confidence intervals (CI) and level of significance for the whole dentition, and for the anterior, premolar and molar regions separately

		D			M			F	
	**OR**	**95% CI**	**P**	**OR**	**95% CI**	**P**	**OR**	**95% CI**	**P**
**All regions**									
Age (per year)	0.996	0.990-1.001		1.008	1.002 - 1.014	**	0.985	0.997 - 0.993	***
Female^a^	1.37	1.15 - 1.63	***	0.60	0.50 - 0.72	***	1.87	1.49 - 2.35	***
Urban^b^	0.71	0.60 - 0.85	***	0.97	0.80 - 1.18		4.18	3.28 - 5.34	***
Angiang^c^	0.98	0.80 - 1.19		1.30	1.04 - 1.62	*	0.41	0.31 - 0.55	***
HCM^c^	0.99	0.80 - 1.23		1.13	0.89 - 1.44		0.88	0.68 - 1.13	
SES high^d^	0.89	0.71 - 1.10		0.84	0.65 - 1.08		1.74	1.36 - 2.21	***
SES low^d^	0.80	0.64 - 1.00		1.39	1.10 - 1.75	**	0.34	0.22 - 0.53	***
									
**Anterior region**									
Age (per year)	0.988	0.981 - 0.995	***	1.024	1.016 - 1.031	***	0.947	0.933 - 0.962	
Female^a^	1.20	0.97 - 1.48		0.67	0.54 - 0.83	***	3.13	2.00 - 4.88	***
Urban^b^	0.70	0.57 - 0.87	***	1.10	0.88 - 1.38		3.78	2.45 - 5.84	***
Angiang^c^	1.11	0.86 - 1.42		1.10	0.85 - 1.42		0.39	0.23 - 0.67	***
HCM^c^	0.74	0.57 - 0.97	*	1.45	1.11 - 1.90	**	0.94	0.60 - 1.46	
SES high^d^	0.77	0.59 - 1.00		1.13	0.85 - 1.50		1.84	1.20 - 2.82	**
SES low^d^	0.78	0.60 - 1.02		1.36	1.04 - 1.78	*	0.63	0.33 - 1.21	
									
**Premolar region**									
Age (per year)	0.984	0.977 - 0.992	***	1.024	1.016 - 1.031	***	0.966	0.951 - 0.981	***
Female^a^	1.52	1.24 - 1.87	***	0.58	0.47 - 0.72	***	2.08	1.37 - 3.16	***
Urban^b^	0.75	0.61 - 0.93	**	1.05	0.85 - 1.30		4.48	2.77 - 7.25	***
Angiang^c^	1.10	0.86 - 1.41		1.12	0.87 - 1.44		0.38	0.22 - 0.66	***
HCM^c^	0.90	0.70 - 1.16		1.32	1.01 - 1.72	*	0.65	0.41 - 1.03	
SES high^d^	1.00	0.77 - 1.30		0.84	0.64 - 1.11		1.54	1.01 - 2.35	*
SES low^d^	1.02	0.78 - 1.33		1.09	0.83 - 1.43		0.22	0.08 - 0.62	**
									
**Molar region**									
Age (per year)	0.979	0.974 - 0.984	***	1.025	1.019 - 1.031	***	0.987	0.979 - 0.996	**
Female^a^	1.21	1.04 - 1.42	*	0.69	0.59 - 0.82	***	1.69	1.32 - 2.17	***
Urban^b^	0.66	0.56 - 0.78	***	1.05	0.88 - 1.24		4.01	3.04 - 5.28	***
Angiang^c^	1.08	0.89 - 1.30		1.14	0.94 - 1.39		0.42	0.30 - 0.59	***
HCM^c^	1.05	0.86 - 1.28		1.07	0.87 - 1.32		0.88	0.67 - 1.16	
SES high^d^	0.91	0.74 - 1.11		0.92	0.74 - 1.14		1.66	1.28 - 2.17	***
SES low^d^	0.91	0.74 - 1.12		1.28	1.03 - 1.58	*	0.24	0.14 - 0.43	***

* = *P *≤ 0.05; ** = *P *≤ 0.01; *** = *P *≤ 0.001

References (OR = 1) respectively: a male, b rural, c Cantho province, d SES middle

Meaning of OR for example for the M_ratio _for the molar region: every year older gives a 2.5% higher chance for missing all non-sound molar teeth. The chance for females compared to males on this event is 31% lower, whereas subject with low SES are estimated to have a 28% higher chance compared to middle SES. Other independent variables showed no statistical significant effects.

### Missing teeth

The percentage of subjects with missing teeth increases from 70% for the youngest age category (20 - 29 yrs.) to 96% for subjects older than 60 years (Table 4). After the age of approximately 30 years the mean number of missing teeth increases almost linearly, amounting to approximately 8 missing teeth per jaw in elderly (Figure 1). As could be expected, the lower anterior region is less prone to have absent teeth than the upper anterior region, however, the mean number of missing teeth at the age of 80 in these regions is similar (mean number of missing anterior teeth in each jaw approximately 2.5; Figure 2). Although increasing with age, the mean number of missing teeth in the upper and lower premolar regions is almost similar for all ages (Figure 3). In contrast to the other regions, the M component in the lower molar region is higher than in the upper for nearly all ages (Figure 4). At the age of 80 on average approximately 2 molars remain in each jaw. Comparison of the M components of the three dental regions (Figures 2, 3 and 4) with the M component of the whole dentition (Figure 1) shows that the M component for the whole dentition is mainly determined by the M component of the molar region. Regression analysis confirms the significant increase of missing teeth with age (Table 5). The effect of aging on M_ratio _is highest in the molar region: every additional year of age gives a 2.5% higher chance for missing all non-sound teeth in this region. In all dental regions females have less missing teeth than males (OR: 0.47 - 0.59; p-values < 0.001). No relationship is found between missing teeth and residence. With regard to province, the chance to have all non-sound teeth missing is highest for subjects from Angiang province (OR: 1.30; p < 0.05), while the chance to miss all non-sound anterior and premolar teeth is highest for subjects from HCM province (OR: respectively 1.45 (*P *< 0.01) and 1.32 (*P *< 0.05)). Subjects with SES low have significantly higher chance for missing non-sound anterior and molar teeth (OR: respectively 1.36 and 1.28; *P *< 0.05).

### Filled teeth

The percentage of subjects with filled teeth is relative low for all age categories (8 to 18%; Table 4). The mean number of filled teeth in this sample is low at all ages in all dental regions (on average 0.1 for each jaw) (Figures 1, 2, 3 and 4). Overall, each additional year of age gives significantly lower chance for having filled teeth (Table 5), except for the anterior region were this effect is also seen but without statistically significance. All other independent variables show influence on F_ratio_, with the highest effect for residence: urban citizens have an approximately 4 times higher chance to have fillings than subjects from rural areas. Furthermore, the regression analysis shows that females more often have fillings than males, subjects from Angiang less often than subjects from Cantho and HCM, and SES high more often than SES middle and SES low.

### Sound teeth

At the age of 20 years, the mean number of sound teeth is approximately 14 in each jaw (Figure 1). This number decreases gradually to approximately 6 in each jaw at the age of 80. The mean numbers of sound teeth are higher in de lower jaw than in the upper jaw at almost all ages for all investigated dental regions except for the molar region, where the mean number of sound teeth is lower in the lower jaw. At older ages the differences between jaws diminish.

### Relative scores for decayed, missing and filled teeth per dental region

Molars showed statistically significant higher risk (paired t-tests) for decay, and missing when compared to premolars and anterior teeth (Table 6). This was most prominent for the lower jaw where molars showed 9.1% more decay than premolars and 11.6% more than anterior teeth. Mean differences for filled are small, being largest for lower molars compared to lower anterior teeth.

**Table 6:** Difference in percentage for molars being decayed, missing or filled as compared to premolars and anterior teeth.

Comparison	Mean difference	95% Confidence Interval	P value
Upper jaw			
D_rel_			
Molars - premolars	4.6	3.8 - 5.4	< 0.001
Molars - anterior teeth	4.5	3.6 - 5.3	< 0.001
M_rel_			
Molars - premolars	1.5	1.4 - 1.6	< 0.001
Molars - anterior teeth	1.5	1.4 - 1.6	< 0.001
F_rel_			
Molars - premolars	0.5	0.3 - 0.8	< 0.001
Molars - anterior teeth	0.3	0.0 - 0.5	0.06
Lower jaw			
D_rel_			
Molars - premolars	9.1	8.3 - 10	< 0.001
Molars - anterior teeth	11.6	10.8 - 12.5	< 0.001
M_rel_			
Molars - premolars	2.8	2.7 - 2.9	< 0.001
Molars - anterior teeth	3.3	3.2 - 3.4	< 0.001
F_rel_			
Molars - premolars	1.6	1.3 - 1.9	< 0.001
Molars - anterior teeth	2.1	1.9 - 2.5	< 0.001

## Discussion

This study aimed to determine the oral health status of adults living in urban and rural areas in Southern Vietnam. Because the inclusion aimed at equal distribution of subjects according to residence, province, gender, and age groups, the sample cannot be considered to be representative for Southern Vietnam. In comparison to the age-group distribution in the population, older subjects are overrepresented in this sample. Age related analyses of the data, however, deal with this overrepresentation. With regard to residence more subjects from rural HCM and more subjects from Angiang were included than can be explained on the basis of their distribution in the population. In addition, the selection of the three provinces, the factories and the communes was not randomly but mainly based on their accessibility. In spite of this, we consider this drawback sufficiently compensated by the large sample size, the random subject sampling, and the high and comparable response rates in the sample subgroups. Nevertheless, the SES-structure of the study sample corresponds with the income-group structure of the population of Southern Vietnam [19]. The same can be concluded with respect to the distributions of educational levels in the sample and the population. In conclusion, while the structure of the study sample is not reflecting the structure of the population, the subjects in this sample may be considered as representative for the population of Southern Vietnam. As a result, age related outcomes at subject level do reflect the oral health status in the population. However, outcomes at sample level are skewed by age and residence and should be interpreted with this in mind. The significant strengths of this study include a large sample size, which provided ample power to evaluate effect modification.

The Kuppuswamy's SES classification and its modifications are widely used for community-based research [17]. As this scale takes both the individual as well as the family socio-economic status into account, it is considered suitable for the Vietnamese situation. Comparison of SES outcomes in this study with governmental data confirms this proposition [19].

The high, with age increasing, prevalence of missing teeth together with the more or less constant and relatively low prevalence of decayed teeth and the constant prevalence of filled teeth indicates that extraction is the most common treatment of decay. This conclusion is in line with earlier findings indicating that tooth extraction is the main treatment in the governmental dental services of Vietnam [11]. Less clear is whether the prevalence of missing teeth is the result of caries only. Data from other studies on reasons of tooth loss indicate that tooth loss due to periodontal involvement is low compared to caries especially for younger ages [20-28]. Additional information from questionnaire and oral examination revealed that caries was the most common reason for tooth loss (91% for molars, 88% for premolars, and 82% for anterior teeth). Tooth loss due to periodontal diseases was respectively 8, 11, and 14% for the different tooth types, which correspondents with findings from another study in Vietnam [29].

As DMF and S_t _data in the present study are based on dentitions comprising of 32 teeth, the missing component is partially determined by congenitally absent and non-erupted third molars, especially for the molar region. The percentage congenitally absent and non-erupted teeth is assessed to be 27.1% for the upper and 21.9% for the lower jaw, with the assessment based on answers from 200 30 to 35 years old subjects with complete dental arches on the question whether they had experienced any extractions. As a result, when estimating the mean number of missing teeth for DMF and S_t_for dentitions comprising of 28 teeth this number should be reduced with approximately 0.25 per jaw. In or exclusion of third molars in the regression analyses does not influence the effects as shown in Table 6.

The higher caries rate for females in the present sample supports the female gender bias for caries prevalence as reported in most epidemiological studies [30].

Urban subjects showed less decay (about 70%) but more fillings (a factor 4) than rural subjects. One might expect that this would result in a lower missing component, which was not the case. One explanation might be that the factor 4 more fillings still reflects a virtually non-existing filling component. Another reason might be the easier access for dental care in urban areas, leading to more interventions (more fillings and more extractions). Few districts of the urban area of HCM (Hochiminh City) are supplied with fluoridated water since 1989. However, the present sample was drawn from districts without water fluoridation. Positive effects reported for districts with water fluoridation [31,32] could therefore not be demonstrated in the present study. The provinces Angiang and Cantho are non-fluoridated.

SES had no influence on decay in this study. This is in line with a systematic review where a strong relationship was found between caries and SES in children and adolescents, but no or only a weak relation in adults [33]. The relationship between low SES and higher prevalence of missing as found in studies from different countries is also seen in Southern Vietnam [34-38]. Like subjects living in urban areas, subjects with higher SES had higher chance for fillings, indicating that restorative interventions are not only determined by easy access but also by affordability.

In this study, molars were statistically significant more often affected by caries, more often missing and less sound than premolars and anterior teeth. This phenomenon has been described earlier in a review [39] summarizing data from numerous studies performed in the 1980s and 1990s and has been confirmed by recent studies [38,40].

Data on the prevalence of missing teeth amongst adult Asians are scarce. A recent systematic review reported that Chinese subjects who are 65 years old had an average of 20 teeth [41]. This figure is in the same range as found in the present study (see Figure 1). Data from European countries show numbers of present teeth in elderly that are a fraction higher: 23.3 teeth in subjects of 60 years, 20.7 teeth in subjects of 70, and 18.4 in subjects of 80 years [42].

This study is the first step in describing the oral health status of Southern Vietnamese adults. The study showed that reduced dentitions are common amongst Southern Vietnamese adults. The 'missing' component of DMFT, however, does not provide information whether these missing teeth are functional in occlusion or not. The impact of having missing teeth can also not be derived from these data. Oral function is less dependent on the number of single teeth present and more dependent on the number and location of occluding pairs. Further research investigating the patterns of tooth loss and their sequels is needed before strategies or treatment protocols such as the shortened dental arch concept can be advised at population level.

## Conclusions

The majority of adults of Southern Vietnam presented a reduced dentition. The combination of low numbers of filled teeth and relative high numbers of decayed and missing teeth indicates that the main treatment for decay is extraction. Molars are more at risk for being decayed or missing than premolars and anterior teeth.

## Competing interests

The authors declare that they have no competing interests.

## Authors' contributions

TCN carried out the data collection and was responsible for preparation of the manuscript. DJW participated in the design and coordination of the study and helped to draft the paper. EMB performed the statistical data analysis. NBT helped with the sampling and coordinated the sampling process. NHJC participated in the study design, supervised the data analyses and interpretation and contributed to the preparation of the manuscript and helped to draft the manuscript. All authors read and approved the final manuscript.

## Pre-publication history

The pre-publication history for this paper can be accessed here:

http://www.biomedcentral.com/1472-6831/10/2/prepub
